# Near Infrared Spectroscopy for Poor Grade Aneurysmal Subarachnoid Hemorrhage—A Concise Review

**DOI:** 10.3389/fneur.2022.874393

**Published:** 2022-04-18

**Authors:** Charles L. Francoeur, François Lauzier, Patrice Brassard, Alexis F. Turgeon

**Affiliations:** ^1^Population Health and Optimal Health Practices Research Unit (Trauma—Emergency—Critical Care Medicine), Centre Hospitalier Universitaire (CHU) de Québec—Université Laval Research Centre, Université Laval, Québec City, QC, Canada; ^2^Department of Anesthesiology and Critical Care, CHU de Québec—Université Laval, Critical Care Division, Québec City, QC, Canada; ^3^Critical Care Medicine Service, CHU de Québec—Université Laval, Québec City, QC, Canada; ^4^Department of Kinesiology, Faculty of Medicine, Université Laval, Québec City, QC, Canada; ^5^Research Center of the Institut Universitaire de Cardiologie et de Pneumologie de Québec, Québec City, QC, Canada

**Keywords:** near infrared spectroscopy, delayed cerebral ischemia, subarachnoid hemorrhage, vasospasm, poor grade aneurysmal SAH, neuromonitoring

## Abstract

Delayed cerebral ischemia (DCI) disproportionately affects poor grade aneurysmal subarachnoid hemorrhage (aSAH) patients. An unreliable neurological exam and the lack of appropriate monitoring leads to unrecognized DCI, which in turn is associated with severe long-term deficits and higher mortality. Near Infrared Spectroscopy (NIRS) offers simple, continuous, real time, non-invasive cerebral monitoring. It provides regional cerebral oxygen saturation (c-rSO_2_), which reflects the balance between cerebral oxygen consumption and supply. Reports have demonstrated a good correlation with other cerebral oxygen and blood flow monitoring, and credible cerebrovascular reactivity indices were also derived from NIRS signals. Multiple critical c-rSO_2_ values have been reported in aSAH patients, based on various thresholds, duration, variation from baseline or cerebrovascular reactivity indices. Some were associated with vasospasm, some with DCI and others with clinical outcomes. However, the poor grade aSAH population has not been specifically studied and no randomized clinical trial has been published. The available literature does not support a specific NIRS-based intervention threshold to guide diagnostic or treatment in aSAH patients. We review herein the fundamental basic concepts behind NIRS technology, relationship of c-rSO_2_ to other brain monitoring values and their potential clinical interpretation. We follow with a critical evaluation of the use of NIRS in the aSAH population, more specifically its ability to diagnose vasospasm, to predict DCI and its association to outcome. In summary, NIRS might offer significant potential for poor grade aSAH in the future. However, current evidence does not support its use in clinical decision-making, and proper technology evaluation is required.

## Introduction

For patients surviving the initial injury of an aneurysmal subarachnoid hemorrhage (aSAH), delayed cerebral ischemia (DCI) deteriorating into cerebral infarction represents the main threat to a favorable outcome (1). Patients with poor grade aSAH [IV and V on the World Federation of Neurosurgeons (WFNS) scale (2)] are the most at-risk (3). They often present an altered level of consciousness, diminishing the reliability of the neurological exam and jeopardizing the application of current consensus DCI definition (4). Compared with clinically apparent DCI, these unrecognized ischemic episodes (5) are associated with worst long-term deficits and higher mortality (6). Transcranial Doppler ultrasonography (TCD), serial vascular or perfusion imaging, electroencephalographic (EEG) monitoring and invasive multimodality monitoring have all been called upon to mitigate these limitations in our ability to detect ischemia. Unfortunately, the evidence supporting their use has been disappointing so far and none has established itself as a reliable modality to circumvent the loss of clinical exam in poor grade aSAH (7).

Near Infrared Spectroscopy (NIRS) technology has been advocated as a solution to this conundrum. It is a simple, portable, continuous, real time, non-invasive monitoring of cerebral oximetry. It should therefore be ideal for timely detection of ischemia and to improve management in poor grade aSAH. The technology has been around for more than 40 years, after it was introduced in 1977 by Jöbsis (8), and subsequently popularized 20 years later by Kirkpatrick (9). Since then, multiple NIRS systems came along, algorithms have improved, a substantial corpus of literature—albeit contradictory and of variable quality—has emerged, and NIRS technology has gained sufficient popularity to be incorporated as a standard of care in many operating theaters and critical care units around the world. However, the quality of evidence supporting it use remains low, and parameters that should influence management, if any, are still unknown.

In this concise review, we lay out the basic concepts required for non-experts to comprehend the technology, we provide a critical appraisal of the possible interpretations and use of the data and its potential impact on poor grade aSAH patients. Closing remarks will review ongoing studies and give some future directions.

## Basic Concepts

The first basic concepts concern optical physics and light attenuation. A NIRS device emits light from a light emitting diode (LED) or a laser in the near infrared range, usually between 700 and 850 nm, and collects what reaches the photodetectors. The loss of light between emitter and detectors represent optical attenuation and is a consequence of two phenomena affecting photon trajectory: absorption and scattering. The absorption is proportional to the concentration of chromophores, which are substances that absorb the light of a specific frequency. In our context, the chromophores of interest are oxyhemoglobin (HbO_2_) and deoxyhemoglobin (HHb). The differential absorption profile should therefore allow the determination of HbO_2_ and HHb content in the region of interest as described by the Beer-Lambert law. There are however competing chromophores and manufacturers must select wavelengths where the absorption spectra of HbO_2_ and HHb are maximally separated and where the overlap with water and melatonin (10) absorption is minimal. The phenomenon of scattering is the other critical variable and the major contributor to attenuation. As light travels through biological tissue, the initial trajectory of the photon is lost, and it is deviated to another direction. The consequences are that some light never reaches the detector, while some reaches it only after being scattered multiple times, traveling a greater distance than the one separating the source and the detector. Deflection is so significant that the path of tissue-reflected photons in the adult head is parabolic rather than in a straight line, explaining why the light source and detectors are placed on adjacent areas of the head. Disentangling the effect of absorption and scattering is one of the crucial roles of the various proprietary algorithms embedded in NIRS devices. They rely on many assumptions, including a fixed arterial: venous ratio (usually 30:70 or 25:75) and a constant scattering, both of which do not reflect what is happening *in vivo*. For example, brain edema creates large shifts in intracranial photon scattering that may profoundly alter readings in an unpredictable manner.

Commercially available oximeters rely on continuous wave data acquisition and spatially resolved spectroscopy (11). The latter is based on the use of multiple detectors, the number of which varies amongst models. As the depth of tissue interrogated is proportional to the distance between emitter and detector, it is assumed that the closest detector receives light that has passed through the scalp, whereas that arriving at the farthest detector has passed through brain tissue. Those distances vary between manufacturers. Multiple detectors therefore allow a gain in spatial resolution and help to mitigate extracranial contamination. This is a considerable weakness of NIRS technology and estimates for the degree of contamination from extracranial tissues range from 7 to 35% (12–14) and vary amongst manufacturers. The biggest impact of spatially resolved spectroscopy, however, is to derive a scaled absolute hemoglobin concentration by combining measures of those closely spaced detectors. That is, the absolute HbO_2_ and HHb contents are unknown, but their relative proportion can be calculated. Percentage of HbO_2_ over total hemoglobin thus provides the cerebral regional oxygen saturation (c-rSO_2_), which is variably coined and abbreviated by manufacturers (rSO_2_, TOI, TCCO, StcO_2_).

Other concepts are about understanding what is being monitored. The probes are normally placed on each side of the forehead, 3 cm above the superciliary line to avoid the frontal sinuses. Consistent positioning is required to minimize variations and obtain reproducible results. As the hemoglobin in larger vessels traps all incident infrared light, surface-detected infrared reflections arise from blood vessels that are <1 mm in diameter (15) and ~1.5 cm under the skin. The presence of intracranial extravascular blood may influence readings because of this infrared photon sequestration. Contrary to pulse oximetry (SpO_2_), NIRS does not require actively flowing and pulsatile flow. It measures a weighted average of arterial, capillary, and venous compartments. C-rSO_2_ thus reflects primarily the small venous compartment of 1.5 cm^3^ of gray matter brain tissue in the territory between the anterior and middle cerebral arteries (16), with some degree of extracranial contamination. True reference values do not exist. Some widely available systems, such as INVOS™, benefit from a high volume of published data allowing the conclusion that for healthy and preoperative adult patients, normal baseline probably ranges between 60 and 75% (17–19). Reports comparing technologies (20, 21) show us that although different NIRS devices provide similar trends, they give different baseline estimates, rendering comparisons difficult. Potential baseline values for aSAH patients are reported in Table 1 (27).

**Table 1:** Baseline regional cerebral oxygen saturation (c-rSO_2_) in aSAH patients.

**NIRS technology**	**Number of patients**	**Poor grade SAH (%)**	**Mean baseline c-rSO_**2**_**	**References**
INVOS™ series (Somanetics, USA)	105	28	60	(22–24)
FORE-SIGHT™ (CAS Medical Systems, USA)	68	24	70	(23, 25)
Nonin series (Nonin Medical, USA)	20	0	64	(26)

## Physiological Data—Oxygenation, Blood Flow, Cerebrovascular Reactivity

C-rSO_2_ is assumed to reflect the balance between cerebral oxygen consumption and supply in the region of interest and therefore to essentially be determined by cerebral metabolic rate of oxygen (CMRO_2_) and oxygen delivery. A pathological decline in c-rSO_2_ might therefore signal arterial hypotension, low cardiac output, systemic hypoxia, hyperventilation, stroke or impending DCI. Conversely, high c-rSO_2_ might be secondary to hyperemia, acidosis, hypoglycemia, high levels of sedation or hypothermia. Convulsions might present as low, high or oscillating c-rSO_2_ (28). How does NIRS-determined c-rSO_2_ compared to other brain oxygen monitoring technologies to detect meaningful events? A few reports evaluated its performance against invasive brain tissue oxygen monitoring (PtiO_2_) and jugular bulb venous oxygenation (SjvO_2_). The comparisons are obviously limited, as PtiO_2_ reflects the partial pressure of dissolved oxygen in interstitial white matter, while SjvO_2_ is the global venous saturation of drained hemispheric blood. Overall, the correlation between NIRS and PtiO_2_ is good. They reflect similar dynamic changes of cerebral oxygen metabolism (29–32), but they vary in their degree and speed of response (33). The sensitivity of NIRS to detect cerebral ischemia, here defined as a PtiO_2_ of <15 mmHg, seems problematic (30), including in the SAH population (34). The same conclusions apply when comparing SjvO_2_ with NIRS. There is a good correlation between modalities (33, 35) and a somewhat lower sensitivity of NIRS to detect significant desaturation (36). Data suggest that NIRS, PtiO_2_ and SjvO_2_ assess different processes that are intimately related, but that crude substitution is not warranted (37).

Several investigators also observed an association between c-rSO_2_ and cerebral blood flow (CBF). Fluctuations in c-rSO_2_ correlate with CBF measurements made by xenon enhanced computed tomography (38, 39) and computed tomography perfusion imaging (40). Ventilation-based CBF manipulations were also used to show the correlation between c-rSO_2_ and CBF as measured with an invasive thermodilution probe (22). Finally, cardiac output augmentation with either dobutamine (41) or milrinone (25) in DCI patients also resulted in a better c-rSO_2_.

More recently, various teams used the information obtained from NIRS monitoring to evaluate cerebrovascular reactivity (42, 43). The reactivity index, variously named tissue oxygenation index (TOx) or cerebral oxygenation index (COx), is calculated as a rolling correlation coefficient between averaged CPP (or MAP) values and the corresponding NIRS signals. It has been validated against pressure reactivity index (PRx) (44) and TCD-derived reactivity index (Mx) (45). It does, however, rely on assumptions that have been regularly challenged (46). Other approaches to evaluate cerebrovascular reactivity have been described, such as those based on frequency-domain analysis, but they have not been applied to our population of interest (47).

If we accept that abnormal c-rSO_2_ reflects a pathological alteration in brain oxygenation, how should it be managed? Most clinicians would suggest investigating plausible and reversible causes as a first step. Correcting obvious systemic physiological derangements such as significant hypoxemia, arterial hypotension, or iatrogenic hyperventilation is also sensible. However, the appropriate management of abnormal c-rSO_2_ in aSAH patients, beyond what was just mentioned, is unclear. The impact of vasopressors, for example, is controversial. Studies have reported a decrease in c-rSO_2_ with vasopressor infusion both in aSAH (48) and healthy patients (49), whereas it improved c-rSO2 values in the first few hours post-cardiac arrest (50). Studies reporting the effect of red blood cell transfusions in neurocritical care patients, a minority of which were aSAH, have also yielded conflicting results (51–53). Controlled hypercapnia, in the range of 50–60 mmHg, had some success to improve c-rSO_2_ in two studies involving aSAH patients (22, 54). This approach, still investigational, is deemed temporary to avoid rebound vasoconstriction. Hyperoxia, targeting supraphysiological levels of arterial partial pressure of oxygen, seems to improve c-rSO_2_ (55), but is also potentially associated with a higher incidence of vasospasm (56), DCI and poor outcome in aSAH (57). It would therefore be inappropriate, based on currently available data, to recommend any specific intervention to attempt correction of abnormal c-rSO2 in aSAH patients.

## Clinical Data—Vasospasm, DCI, Prognosis

The ultimate objective behind adequate monitoring of cerebral oxygenation, perfusion and cerebrovascular reactivity is early detection of secondary brain injury and management guidance to improve patient outcomes. Vasospasm detection, DCI prediction, and prognostication have been specifically evaluated in the aSAH population. Comparisons between TCD and commercially available NIRS seem to support a moderate degree of correlation between the two modalities (58). A decrease of more than 12% in c-rSO_2_ from the baseline was associated with a better predictive value than same-side TCD using the traditional threshold value of 200 cm/s to detect severe vasospasm on CT angiography (59). More interestingly, the same 12% cut-off yielded a sensitivity of 94% (95% CI: 73–99%) and a specificity of 71% (95% CI: 53–85%) to detect DCI (59). Another study on 24 patients presenting with poor grade aSAH reported a sensitivity of 86% (95% CI: 67–98%) and specificity of 86% (95% CI: 67–96%) for DCI detection using a greater than 15% decrease in c-rSO_2_ (60).

Others took a different approach and evaluated the association between impaired cerebrovascular reactivity and DCI. Using an index of |*R*| ≥ 0.5 on either side as a definition for impaired cerebrovascular reactivity, and the consensus definition for DCI, one group reported odds of DCI of 36 (95% CI: 6–211%) when impaired cerebrovascular reactivity was present (23). Another investigator used TOx and found that impaired cerebrovascular reactivity on the side contralateral to the aneurysm was significantly more frequent in the DCI group than in the non-DCI group (58 vs. 16%, *p* = 0.014) and was associated with an OR of DCI of 19 (95% CI: 1.2–320) (24). Using a threshold of 0.07 conferred TOx a sensitivity of 58% and a specificity of 91% to predict DCI.

Only three studies have examined the relationship between c-rSO_2_ and outcome in aSAH patients. In one study, 163 aSAH patients were monitored between day 5 and 10 after aneurysm rupture (26). Using the definition of cerebral desaturation of <50% for 30 min on either side, and poor outcomes as a mRS of 4–6, it was observed that cerebral desaturation was independently associated with poor functional outcomes at 3 months (OR 2.72, 95%CI 1.02–7.20) but not at 12 months. In a small study of 38 patients using a definition of cerebral desaturation of <60% for at least 30 min on either side, patients with an unfavorable outcome spent more time with a cerebral desaturation than those with a good outcome (5 h 43 vs. 1 h 47, *p* = 0.02) (61). Patients with episodes of cerebral desaturation lasting more than 2 h were at much higher risk of poor short-term outcomes than those without [OR 15.4 (95% CI: 1.1–214.2%)]. Another small study of 31 patients evaluated the association between NIRS-based cerebrovascular reactivity indices and optimal blood pressure with functional outcomes at 3 months and defined unfavorable outcome as ≥ 3 on the mRS scale (44). In this study, preserved cerebrovascular reactivity, as defined by a negative or near-zero TOx was associated with good functional outcomes at 90 days (OR, 2.5; 95% CI, 1.3–4.8), including after adjustment for age, WFNS and DCI. Using NIRS-derived optimal blood pressures, %time outside the limits of autoregulation was significantly associated with poor 90-day outcomes (OR, 1.9; 95% CI, 1.3–2.9) and deviation from NIRS-derived autoregulatory limits predicted poor 90-day outcomes with high sensitivity (0.82; 95% CI, 0.67–0.98) and specificity (0.88; 95% CI, 0.76–1.00) (34). The studies before mentioned are observational. No NIRS-based intervention study has been conducted in SAH patients with long term functional outcome as a primary outcome. There is no data to support specific NIRS-based management recommendations to improve outcome in this population.

## Discussion and Perspective

Despite widespread availability for more than two decades and obvious user-friendly characteristics, the role of NIRS in aSAH management is still ill-defined. The technology possesses obvious advantages: simple, safe, non-invasive, continuous. Unfortunately, limitations are significant. One is the important differences amongst devices: types and amounts of light sources, wavelengths used, the distance between the emitters and detectors, the number of detectors, arterial:venous ratio assumptions, underlying proprietary algorithms, reported normal baseline values, degree of extracranial contamination. All these affect data accuracy and interpretation, complexifies the adoption of conclusions obtained from one device to another and is akin to work with uncalibrated instruments. Technical problems, although rarely reported, should also be considered. In some studies, up to 20% of the readings were non-valid (34, 62). Competition for space on the forehead for electroencephalography and lack of long-term adherence of probes because of sweat are also potentially common (62). Frontal hematoma, wounds on the forehead and deficient NIRS signal are sometimes used as exclusion criteria (30) and bifrontal decompressive craniectomies prevents usual NIRS placement and interpretation (53). This review only covered commonly used NIRS technologies at the bedside, but other devices rely on time domain (63) and frequency domain spectroscopy (64) rather than continuous wave spectroscopy. Some also employ hybrid technologies such as ultrasound tagged near-infrared spectroscopy (65) or diffuse correlation spectroscopy and diffuse optical spectroscopy (66) to provide relative estimates of CBF changes. However, promising these technological advances might sound, none was proven superior for clinical applications in aSAH.

The lack of a normal range of c-rSO_2_ for neurocritical care patients, especially aSAH, is troublesome. The lack of consensus regarding a lower limit of NIRS-derived c-rSO_2_ values, serving as an intervention threshold, is even more problematic. Various thresholds, duration, variations from the baseline, asymmetries, cerebrovascular reactivity indices and ischemic burdens have been described. None has established itself as a significant physiological or clinical marker that should modify management and the authors would be hard-pressed to suggest a specific one. The overall low quality of the clinical research is important to highlight. Published data on the use of NIRS in aSAH since 1998 are all but one unblinded, uncontrolled, single center observational studies, a third of which included fewer than 15 patients. Consensus definition of DCI and cerebral infarction are used in less than a third of the studies, and 90 days outcome are reported in <25%. Poor-grade aSAH, the population most susceptible to benefit from such a monitoring, is vastly underrepresented.

Conducting research on DCI in poor grade aSAH is difficult. DCI is a complex, elusive and evolving entity lacking a gold standard in patients without a proper neurological exam. No evidence-based treatment exists. Our understanding of brain physiology, including oxygenation, perfusion and autoregulation, is incomplete and the impact of a monitoring strategy on clinical decision-making is more complex than simply assessing accuracy. Nonetheless, NIRS technology should be submitted to rigorous evaluation and assessment. We suggest that clinical investigators focus on pragmatic, bedside-applicable hypotheses rather than exploratory ones. The heterogeneity of “ischemic” indices already hinders clinical research tremendously. The population of poor grade aSAH should be targeted. Observational studies are to be prospective and blinded, and intervention studies should be properly conducted, pragmatic and multicenter randomized clinical trials. Outcomes should include cerebral infarction on MRI as a surrogate for DCI and 90 days or more functional and quality-of-life outcomes using a validated scale, aligned with published common data elements (67). As no gold standard exists for monitoring poor grade aSAH, comparison with other monitoring tools seems futile. At least two upcoming trials might help to shade some light on the use of NIRS in aSAH patients. One is the NeurO_2_ study, a prospective, blinded, multicenter observational study that will recruit close to 300 TBI and aSAH patients, monitoring them with NIRS and evaluating the outcome at 6 months using the Glasgow Outcome Scale extended and EQ-5D-5L (68). The other one is an interventional, multicenter, single-blinded, randomized clinical trial aiming to enroll 150 aSAH patients to evaluate NIRS-directed optimal cerebral perfusion pressure on Glasgow outcome scale at 6 months (69).

Improving outcomes in aSAH patients is intrinsically associated with earlier detection and treatment of DCI, preventing evolution toward cerebral infarction and the associated sequelae. Poor grade aSAH are at high risk of DCI, their clinical examination is suboptimal, and monitoring alternatives are limited. NIRS technology profile is promising, but current evidence does not support its use to guide management in this population. High-quality research is urgently needed.

## Author Contributions

CF contributed by reviewing the current literature and writing the manuscript. FL, PB, and AT contributed by reviewing the manuscript and making substantive intellectual contributions. All authors read and approved the manuscript.

## Conflict of Interest

The authors declare that the research was conducted in the absence of any commercial or financial relationships that could be construed as a potential conflict of interest.

## Publisher's Note

All claims expressed in this article are solely those of the authors and do not necessarily represent those of their affiliated organizations, or those of the publisher, the editors and the reviewers. Any product that may be evaluated in this article, or claim that may be made by its manufacturer, is not guaranteed or endorsed by the publisher.
